# Adipose tissue-derived stem cell exosomes enhance skin barrier function and show exploratory associations with the skin mycobiome in aging skin

**DOI:** 10.71150/jm.2603020

**Published:** 2026-06-30

**Authors:** Bo-Yun Choi, Hye-Jin Kim, Myeong Jae Kim, Yoon Jin Roh, Ji Yeon Hong, Kui Young Park, Woo Jun Sul

**Affiliations:** 1 Department of Systems Biotechnology, Chung-Ang University, Anseong 17546, Republic of Korea; 2 Department of Dermatology, Chung-Ang University College of Medicine, Seoul 06973, Republic of Korea

**Keywords:** skin microbiome, aging skin, HACS, fungal microbiome

## Abstract

Skin aging increases transepidermal water loss (TEWL), reduces elasticity, and perturbs the skin microbiome. Adipose tissue-derived stem cell exosomes (ASCE) show regenerative potential; however, their clinical effects on skin physiology and microbiome remain unclear. We conducted a split-face, randomized controlled trial in 16 adults aged ≥ 40 years with visible facial aging. One facial side received ultrasound-assisted transdermal delivery of a human ASCE-containing solution (HACS), whereas the other side received normal saline, at two-week intervals for three sessions. Biophysical outcomes (TEWL, stratum corneum hydration, and elasticity parameters R2/R5/R7) were assessed at baseline and week 2, 4, and 8. Wrinkles, pigmentation, and sebum levels were quantified using Mark-Vu imaging, and the Physician’s Global Aesthetic Improvement Scale (PGAIS) and patient satisfaction assessment scores were recorded. Skin swabs from ten participants were subjected to 16S rRNA and ITS1 sequencing. HACS treatment significantly reduced TEWL (*p* = 0.006 at week 2; *p* = 0.009 at week 8) and increased hydration (*p* < 0.001 at all time points) with a significant increase in elasticity (R2/R5/R7 values, *p* < 0.001). Both the PGAIS and patient satisfaction scores were significantly higher on the experimental side. Bacterial α/β-diversity remained largely unchanged, and no bacterial taxa remained significantly associated with skin parameters after FDR correction. In contrast, several fungal taxa showed significant positive associations with skin parameters after FDR correction, detectable only on the HACS-treated side. No significant adverse events were observed. HACS improved barrier function, elasticity, and aesthetic outcomes, whereas microbiome analyses suggested a modest fungal response associated with treatment-related skin changes in aging skin.

## Introduction

Skin aging is a complex biological process influenced by intrinsic factors, such as chronological aging (Wong and Chew, 2021), and extrinsic factors, including ultraviolet (UV) radiation (Gentile and Garcovich, 2021), pollution (Martic et al., 2022), and oxidative stress (Kammeyer and Luiten, 2015). Skin aging is characterized by structural and functional deterioration (Wong and Chew, 2021), leading to visible signs of wrinkles, loss of elasticity, uneven pigmentation, and dehydration. Beyond aesthetic concerns, skin aging can affect psychological well-being (Szlávicz et al., 2024), contributing to reduced self-confidence, social withdrawal, and depressive symptoms. Consequently, there is a growing demand for effective anti-aging interventions that not only improve skin appearance but also restore skin function at the cellular and molecular levels.

Recent advancements in regenerative medicine have led to the development of skin boosters (Haddad et al., 2022; Yi et al., 2024), which are a novel class of biostimulatory agents that enhance skin rejuvenation by delivering bioactive substances directly into the dermal layer. Among these, exosome-based therapies have gained significant attention owing to their ability to facilitate intercellular communication and promote tissue regeneration (Domaszewska-Szostek et al., 2025; Lei et al., 2025). Exosomes are extracellular vesicles (30–200 nm in diameter) released by various cell types, including adipose tissue-derived stem cells (ASCs). ASC-derived exosomes (Shin et al., 2025) have been shown to consist of a rich array of growth factors, cytokines, lipids, and microRNAs, which modulate inflammation and senescence, enhance wound healing and rejuvenation, and minimize risks associated with traditional stem cell-based products. A recent study has reported that an ASC-exosome-mediated increase in sphingosine-1-phosphate (S1P) suppressed inflammatory cytokine production in a skin inflammatory model of human keratinocytes (Shin et al., 2025). These properties make them promising candidates for anti-aging interventions. Clinically, a randomized split-face trial demonstrated that combining microneedling with ASC-exosomes (HACS) resulted in significantly greater improvements in wrinkles, elasticity, moisture content, and pigmentation after 12 weeks compared with microneedling alone (Park et al., 2023).

Exosome-based products have demonstrated potential in areas such as atopic dermatitis (Cho et al., 2018; Shin et al., 2020) and wound healing (Lee et al., 2023), their efficacy in skin aging and skin microbiome modulation remains underexplored. The skin microbiome encompasses a complex community of commensal bacteria, fungi, viruses, archaea, and mites, which together establish diverse ecological niches characterized by varying densities and compositions (Boxberger et al., 2021). This microbial ecosystem plays a pivotal role in maintaining skin homeostasis by supporting the barrier functions of the skin, including physical, chemical, and immunological defenses (Lee and Kim, 2022; Lee et al., 2025). Emerging research suggests that the skin microbiome plays a crucial role in maintaining skin homeostasis and regulating aging process (Garlet et al., 2024; Han and Kim, 2024; Zhou et al., 2023). Studies employing metagenomic and 16S rRNA sequencing have revealed that skin aging is associated with alterations in microbial diversity, which affect lipid metabolism (Shibagaki et al., 2017), ceramide synthesis (Woo and Kim, 2024), and oxidative stress response (Ratanapokasatit et al., 2022). Furthermore, microbial enzymes involved in protein glycation have been implicated in the accumulation of advanced glycation end products (AGEs), which contribute to collagen degradation (Pageon et al., 2014) and skin laxity (Mohiuddin, 2019). Given these findings, it is plausible that exosome-based therapies not only restore skin hydration and elasticity but also modulate the skin microbiome, potentially mitigating age-related dysbiosis.

This study aimed to evaluate the anti-aging effects of HACS, with a particular focus on its impact on skin barrier function, hydration, elasticity, and the microbiome. Using a split-face randomized controlled trial design, we sought to assess the clinical efficacy of HACS compared to a saline control by measuring key dermatological parameters such as transepidermal water loss (TEWL), skin hydration (measured via corneometry), and skin elasticity (measured via cutometry). Additionally, a subset of participants underwent skin microbiome analysis to determine whether exosome treatment influences the microbial composition and diversity. By integrating clinical dermatology with microbiome research, this study aimed to explore potential links between HACS, skin physiology, and the skin microbiome. The findings not only provide scientific validation for exosome-based anti-aging therapies but also offer insights into their cosmetic and cosmeceutical potential, advancing microbiome-informed strategies to address skin aging.

## Materials and Methods

### Study design and participants

A total of 16 healthy adult volunteers (≥ 40 years of age) with visible signs of facial skin aging were enrolled in this study. All participants met the inclusion criteria and provided written informed consent before participation. This study was conducted at the Chung-Ang University Hospital between March 2024 and February 2025. This study was approved by the Institutional Review Board of the Chung-Ang University Hospital (IRB No. 2402-019-591), and all methods were performed in accordance with the relevant guidelines and regulations. The study employed a split-face, randomized controlled design, in which each participant’s face was divided along the midline. One side was randomly assigned to receive the investigational product HACS (ExoCoBio Inc., Korea) (Park et al., 2023) containing ASC-exosomes, whereas the contralateral side received 0.9% normal saline as a control. Both treatments were delivered via ultrasound-assisted transdermal application at 2-week intervals for three sessions over 8 weeks. The participants were instructed to use a standard cleanser and moisturizer provided by the investigators throughout the study period. The inclusion criteria were as follows: (1) male or female participants 40 years or older with good overall health; (2) willingness to comply with all study procedures and visit schedules; and (3) agreement to abstain from any cosmetic facial treatments, such as filler injections, photorejuvenation, chemical peels, or dermabrasion during the study period. The exclusion criteria were as follows: (1) planned facial cosmetic procedures during the study; (2) presence of acute facial dermatitis or active skin infection; and (3) any condition deemed by the investigator to render the subject unsuitable for study participation.

### Preparation and characterization of HACS

Adipose stem cell-derived exosomes (ASCE) are small extracellular vesicles isolated from adipose stem cell-conditioned medium using tangential flow filtration (TFF)-based ExoSCRTTM technology, as previously described (Lee et al., 2024; Shin et al., 2025). Briefly, conditioned medium was passed through a 0.22-μm membrane to remove cells, cell debris, microvesicles, and apoptotic bodies, followed by concentration and purification using a 500-kDa molecular weight cut-off filter. In prior studies, vesicles prepared using this platform were characterized according to the MISEV2018 recommendations by transmission electron microscopy/cryo-transmission electron microscopy, nanoparticle tracking analysis, and flow cytometric confirmation of CD9, CD63, and CD81, with low or residual levels of negative markers such as calnexin and cytochrome C. HACS refers to a human adipose tissue-derived stem cell exosome-containing solution formulated with ASCE and supplementary ingredients for dermatologic application. In the present study, the investigational product HACS (clinical formulation: ASCE +SRLV; ExoCoBio Inc., Korea) was administered by ultrasound-assisted transdermal application according to the study protocol. Previous dermatologic studies have supported the feasibility of ASCE-based formulations for facial skin application (Cho et al., 2018; Kwon et al., 2020).

### Assessment of skin biophysical and clinical parameters

Clinical evaluations were conducted at baseline and at 2, 4, and 8 weeks after the initial treatment. Objective biophysical parameters were assessed using standard instruments: TEWL was measured using a tewameter (TM-300; Courage & Khazaka Electronic GmbH, Germany), stratum corneum hydration was evaluated using a corneometer (CM-825; Courage & Khazaka), and skin elasticity (parameters R2, R5, and R7) was assessed using a cutometer (MPA580; Courage & Khazaka). Facial skin characteristics including wrinkle depth, pigmentation, sebum level, and brightness, were quantitatively analyzed using Mark-Vu facial analysis system (PSI Plus Co., Ltd., Korea), which captures multi-angle images under four light sources (standard, polarized, specular, and UV) to assess various dermatological features. DSLR photography was performed under consistent lighting and positioning conditions for visual documentation. Additionally, the Physician’s Global Aesthetic Improvement Scale (PGAIS) was used to evaluate clinical improvement, and subjective treatment satisfaction was recorded using patient-reported outcome questionnaires at each visit.

### Skin sampling and DNA extraction

Skin swab samples were collected from enrolled participants, and 10 participants were included in the final microbiome analysis set. Samples were obtained by swabbing a standardized 4 cm² area on the cheek using a sterile cotton swab, followed by gentle pressure for 2 min and 30 s to ensure sufficient microbial yield without causing skin irritation. The swab samples were stored at −80°C until DNA extraction. Genomic DNA was extracted using the PureLink^®^ Genomic DNA Mini Kit (Invitrogen, Life Technologies, USA) in combination with a bead-beating method optimized for skin microbiome samples. Swabs were first incubated in a lysozyme digestion buffer (20 mM Tris, pH 8.0, 2 mM EDTA, 1.2% Triton X-100, lysozyme at 20 mg/ml) at 37°C for 1 h. Following this, 5 mm stainless steel beads and proteinase K were added to each sample. The samples were then subjected to bead beating using a Bead Beater 16 (BioSpec Products Inc., USA) for 1 min, followed by incubation at 55°C for 1 h. After pre-treatment, DNA was extracted according to the manufacturer’s protocol for gram-positive bacterial cell lysis. The concentration and purity of the extracted genomic DNA were assessed using NanoDrop 2000 spectrophotometer (Thermo Fisher Scientific Inc., USA). All microbiome samples were processed using the same collection, DNA extraction, amplification, and sequencing workflow. Samples were excluded only if they failed predefined sample availability or downstream sequencing quality criteria. No samples were excluded solely on the basis of unusual taxonomic composition.

### Polymerase chain reaction (PCR) and sequencing

To analyze the bacterial and fungal communities, we amplified the respective target regions using PCR. For bacteria, the full-length 16S rRNA gene, encompassing the V1–V9 hypervariable regions, was amplified for sequencing on the PacBio Revio platform. PCR amplification of the bacterial 16S rRNA gene was performed under the following conditions: initial denaturation at 95°C for 3 min, followed by 25 cycles of denaturation at 95°C for 30 s, annealing at 57°C for 30 s, and extension at 72°C for 60 s, with a final extension at 72°C for 5 min. For fungi, the ITS1 region was amplified using the primer pair ITS1 18S-F (GTAAAAGTCGTAACAAGGTTTC) and ITS1 5.8S-1R (GTTCAAAGAYTCGATGATTCAC). The PCR conditions were as follows: initial denaturation at 95°C for 3 min, followed by 33 cycles of denaturation at 95°C for 30 s, annealing at 55°C for 30 s, and extension at 72°C for 30 s, with a final extension at 72°C for 5 min. Bacterial 16S rRNA amplicons were sequenced using the PacBio Revio platform. SMRTbell libraries were prepared, and high-quality consensus reads were obtained using Circular Consensus Sequencing. For the fungal community analysis, ITS1 amplicons were sequenced using the Illumina MiSeq platform.

### Bacterial and fungal community analysis

Amplicon Sequence Variants (ASVs) were generated, and microbial diversity was calculated using the Quantitative Insights Into Microbial Ecology 2 (QIIME2) pipeline (Bolyen et al., 2019). For bacterial data, primer and adapter sequences were removed using cutadapt prior to importing the sequences into QIIME2 (Martin, 2011). Denoising and base error correction were performed to generate ASVs and a feature table. Taxonomic classification was performed using the assignTaxonomy function from the DADA2 package in R (Callahan et al., 2016), based on the RDP database (Wang and Cole, 2024). The mitochondrial- and chloroplast-derived ASVs were filtered. ASV alignment and phylogenetic tree construction were performed using the align-to-tree-mafft-fasttree plugin in QIIME2. For the fungal data, paired-end sequences were processed using the DADA2 plugin in QIIME2. Primer and adapter sequences were removed, and the reads were merged with a minimum quality score threshold (Q-score) of 30. Taxonomic assignment of fungal ASVs was performed using the UNITE database (Abarenkov et al., 2010). Beta diversity was calculated to evaluate differences in bacterial and fungal community compositions between groups using both unweighted and weighted UniFrac distances. To assess correlations between microbial composition and clinical variables, Spearman’s rank correlation was calculated using the cor.test in R.

### Statistical analysis

To assess differences in clinical parameters between the control and experimental sides at each visit, we used the Wilcoxon signed-rank test, taking into account the paired split-face design. Differences in alpha diversity between paired samples were also evaluated using the Wilcoxon signed-rank test. Differences in microbial community composition were assessed by permutational multivariate analysis of variance (PERMANOVA), and differences in beta-dispersion were evaluated using a dispersion test in R. Correlations between clinical parameters and bacterial or fungal taxa were analyzed using Spearman’s rank correlation. To account for multiple testing in the correlation analyses, *p*-values were adjusted using the false discovery rate (FDR) method. Statistical significance was defined as *p* < 0.05 for group comparisons and *q* < 0.05 for FDR-adjusted correlation analyses.

## Results

### Changes in skin biophysical parameters

**Fig. 1.**
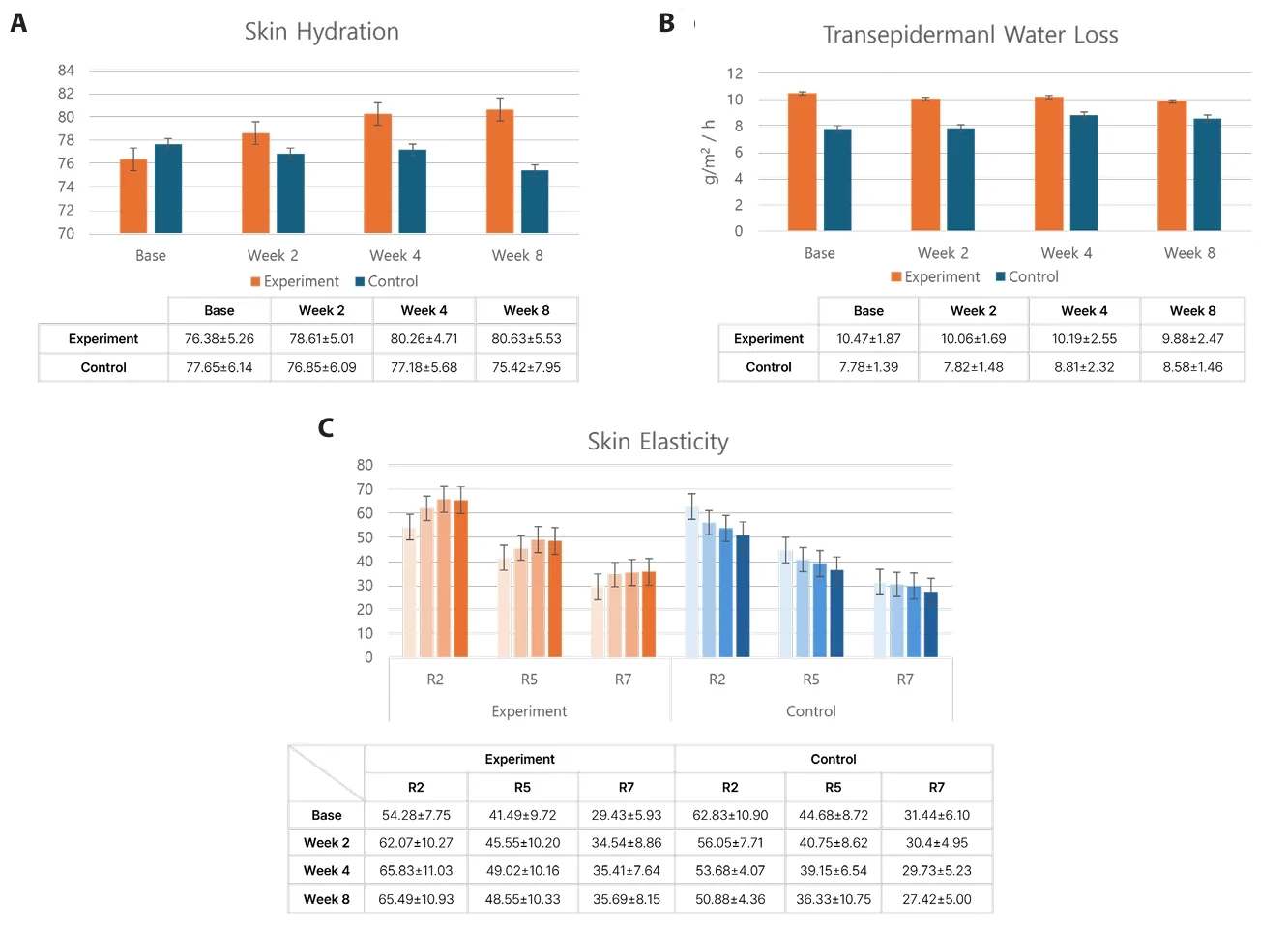
Clinical parameters assessed at each follow-up visit: (A) Stratum corneum hydration measured using Corneometer^®^, (B) transepidermal water loss (TEWL) measured using Tewameter^®^, and (C) skin elasticity measured using Cutometer^®^.

The moisture content of the stratum corneum, measured using a corneometer (CM-825; Courage & Khazaka), increased significantly at weeks 2, 4, and 8 relative to baseline on the experimental side (*p* = 0.014, 0.01, and 0.006, respectively) (Fig. 1A). In contrast, the control side showed a decreasing but statistically insignificant trend in hydration over the same period.

TEWL, measured using a tewameter (TM-300; Courage & Khazaka), significantly decreased in the experimental side at week 2 (*p* = 0.006) and showed a decreasing trend at week 4 and 8 (Fig. 1B). Conversely, the control side showed a gradual increase in TEWL, which was statistically significant at week 8 (*p* = 0.02).

Skin elasticity, measured via cutometer parameters (R2: gross elasticity, R5: net elasticity, R7: skin firmness), showed consistent and significant improvement across all post-treatment time points in the experimental side (*p* < 0.01 for all measures) (Fig. 1C). In contrast, the control side showed a significant decrease in R2 at week 2 and 8 compared to baseline (*p* = 0.032 and *p* = 0.01, respectively). R5 also decreased significantly at week 8 (*p* = 0.006), whereas R7 showed a decreasing but statistically insignificant trend at week 2, 4, and 8.

Additional skin parameters, including pore size, wrinkle depth, freckle count, sebum level, and pigmentation, were measured using the Mark-Vu system. No significant differences were observed in the majority of parameters; however, wrinkle depth was significantly reduced at week 8 in the experimental side compared to baseline (*p* = 0.04) (see Table S1).

### Changes in subjective and physician-assessed clinical parameters

**Fig. 2.**
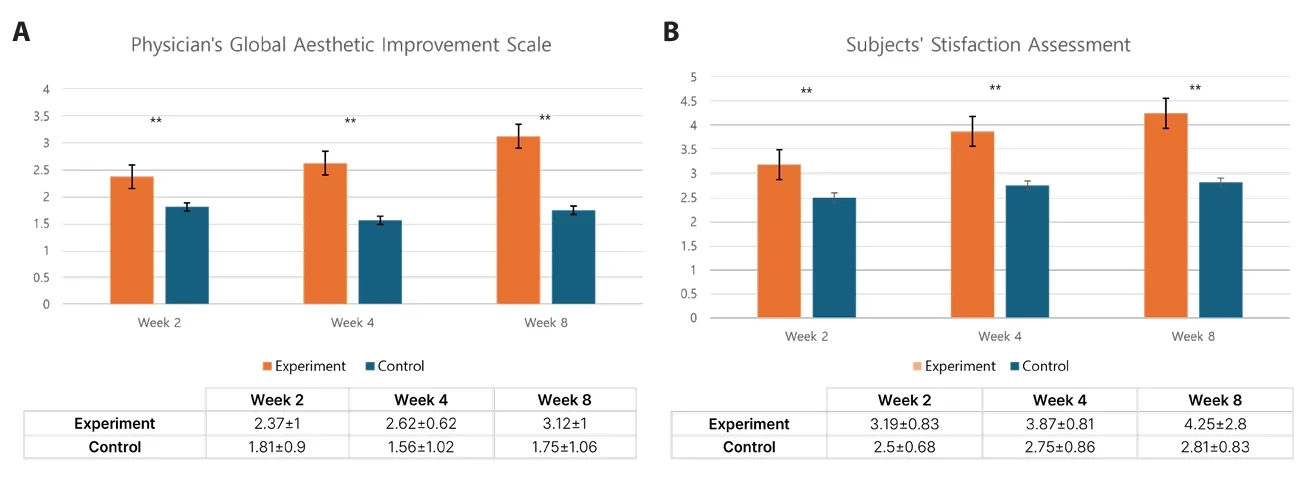
Physician-assessed Global Aesthetic Improvement Scale (PGAIS) and subject-reported satisfaction assessment. (A) Investigator-rated PGAIS at week 2, 4, and 8. (B) Subject satisfaction assessment scores recorded at every visit.

The average score on the PGAIS was higher in the experimental side than in the control side at week 2, 4, and 8 (Fig. 2A). Notably, some participants in the experimental side showed visible improvements, such as reduced crow’s feet wrinkles and brighter skin tone, as observed by the physician. Similarly, the subjects’ satisfaction assessment scores were higher in the experimental side than in the control side across all time points (Fig. 2B). The average satisfaction score on the experimental side increased over time.

### Changes in skin microbiome following HACS treatment

When bacterial communities were compared across visits (baseline and weeks 2, 4, and 8) within each side, no significant differences in community composition were observed (Fig. S1A; PERMANOVA: unweighted UniFrac, control *p* = 0.771 and experimental *p* = 0.222; weighted UniFrac, control *p* = 0.938 and experimental *p* = 0.976). Likewise, bacterial community composition did not differ significantly according to clinical parameters, including PGAIS and subjects’ satisfaction assessment scores (Fig. S2; all PERMANOVA *p* > 0.05). The bacterial composition profiles of all participants during the study period are shown in Fig. S3A. Marked inter-individual variation was observed, with participant no. 10 showing a relatively high abundance of unclassified *Neisseriaceae*, whereas participants 11 and 13 were characterized by a predominance of *Cutibacterium acnes*. Given this substantial inter-individual variability, no significant differences were identified in bacterial alpha-diversity (data not shown) or beta-diversity between sides.

In contrast, fungal composition was relatively consistent across participants. Fungal taxa belonging to the genus *Malassezia*, including *Malassezia restricta*, *Malassezia* sp., and *Malassezia globosa*, were detected in all participants (Fig. S3B). No significant differences in fungal alpha-diversity (data not shown) or beta-diversity were observed overall. Similarly, fungal community composition did not differ significantly across visits within either side (Fig. S1B; unweighted UniFrac: control PERMANOVA *p* = 0.382 and experimental *p* = 0.490; weighted UniFrac: control PERMANOVA *p* = 0.957 and experimental *p* = 0.772), and no significant differences in dispersion were detected in these comparisons. Fungal community composition also did not differ significantly according to PGAIS or subjects’ satisfaction assessment scores (Fig. S4; all PERMANOVA *p* > 0.05). Although significant differences in dispersion were observed in the unweighted UniFrac analysis for physician-assessed improvement on the control side (*p* = 0.025) and for subject-reported satisfaction on both the control side (*p* = 0.008) and the experimental side (*p* = 0.034), no corresponding significant differences in overall community composition were detected by PERMANOVA.

### Identification of microorganisms associated with clinical parameters

We performed correlation analyses to identify microbial taxa associated with skin parameters. No bacterial taxa remained significantly associated with skin parameters after FDR correction (all *q* > 0.05). Several nominal associations were observed at the raw *p*-value level; however, these did not remain significant after multiple-testing correction and are therefore shown in Fig. S5.

**Fig. 3.**
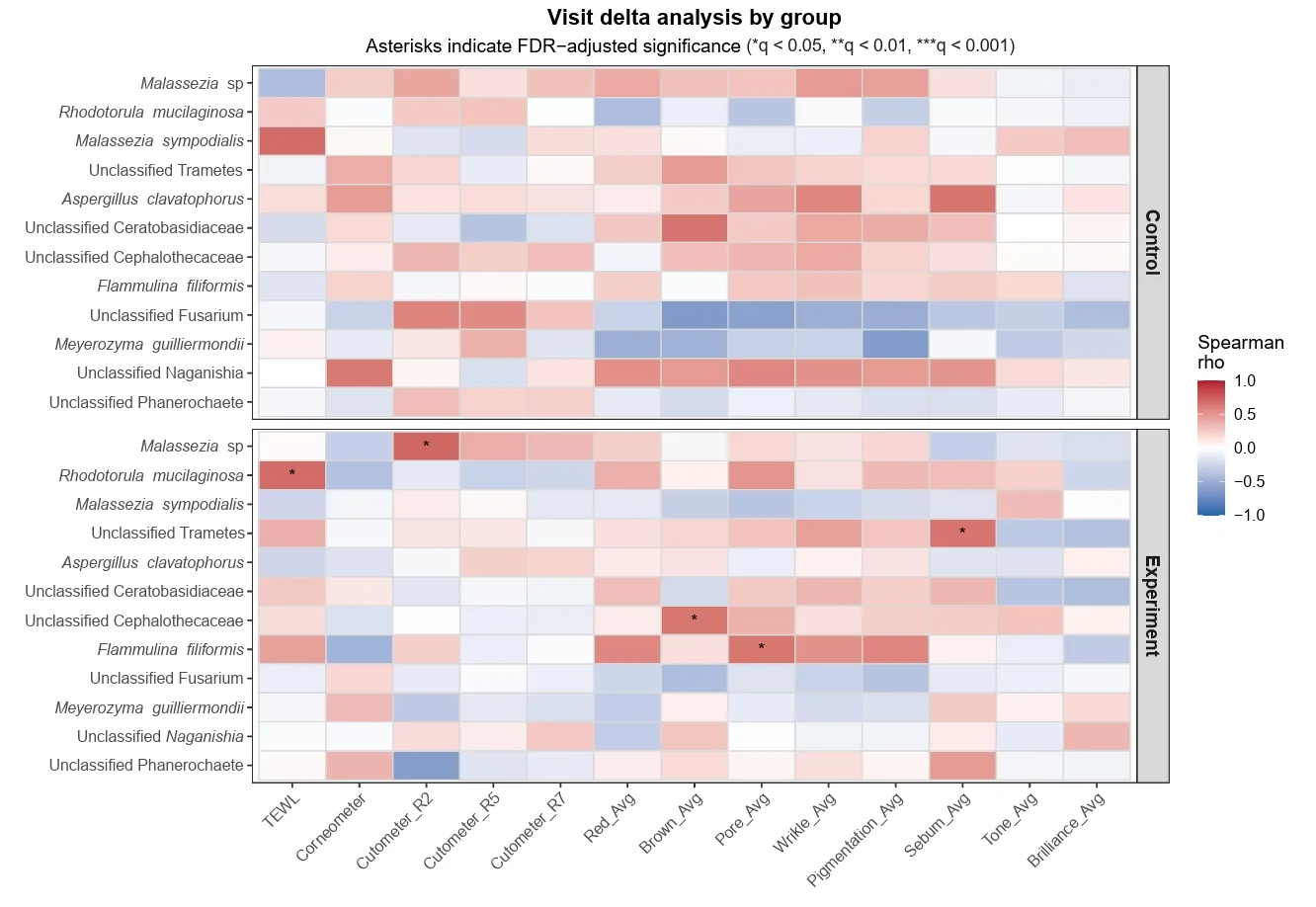
Visit delta analysis of fungal taxa and skin physiological parameters by group. Spearman correlation heatmap showing associations between changes in fungal taxa abundance and changes in skin physiological parameters from baseline (V1) to subsequent visits. The upper panel represents the control side, and the lower panel represents the experimental side. Rows indicate fungal taxa, and columns represent changes (Δ) in skin parameters, including transepidermal water loss (TEWL), corneometer values, cutometer parameters (R2, R5, and R7), and additional skin metrics (redness, brownness, pore, wrinkle, pigmentation, sebum, tone, and brilliance). Color scale indicates Spearman’s correlation coefficient (rho), with red representing positive correlations and blue representing negative correlations. Asterisks denote statistically significant associations after false discovery rate (FDR) correction (*q < 0.05, **q < 0.01, ***q < 0.001).

By contrast, several fungal taxa remained significantly associated with skin parameters after FDR correction (Fig. 3). *Malassezia* sp., *Rhodotorula mucilaginosa*, unclassified Cephalothecaceae, *Flammulina filiformis*, and unclassified *Trametes* showed significant positive correlations with Cutometer R2, TEWL, brown pigmentation, pore, and sebum parameters, respectively. Notably, these significant fungal associations were identified only on the experimental side in the present dataset.

## Discussion

In this study, we found beneficial effects of HACS on key skin parameters, including hydration, elasticity, and barrier function. HACS treatment significantly improved stratum corneum hydration, reduced TEWL, and increased skin elasticity across all measured parameters (R2, R5, and R7). These objective biophysical improvements were further supported by higher PGAIS and patient satisfaction assessment scores in the experimental side than in the control side. Together, these findings support the potential of HACS as a non-invasive skin rejuvenation strategy.

No significant changes were observed in the overall bacterial or fungal community structure on either the experimental or control sides, and both communities generally appeared to remain stable throughout the study period. In particular, bacterial composition showed substantial inter-individual variation, with some participants exhibiting distinct community profiles that were maintained consistently across visits regardless of treatment. This relative stability may reflect the resilience of the skin bacterial ecosystem to short-term intervention (Jones et al., 2021; SanMiguel et al., 2018; Saville et al., 2022). From a skin health perspective, such stability may be viewed favorably, as improvement in skin biophysical parameters was not accompanied by evidence of bacterial dysbiosis. In other words, while HACS was associated with improvement in several skin parameters, it did not appear to disrupt the resident bacterial microbiome.

In contrast, within the fungal dataset, several specific fungal taxa showed significant associations with skin parameters after FDR correction. Notably, these associations were detected only on the experimental side in the present dataset. Although no strong community-wide shift was observed, this pattern raises the possibility that fungal taxa may be more responsive than bacterial taxa to local physiological or microenvironmental changes in the skin (Nguyen and Kalan, 2022). One possible explanation is that HACS treatment influenced barrier status and related skin conditions, including hydration, sebum, and pigmentation-associated features, and that certain fungal taxa tracked these changes more sensitively than bacterial taxa. However, these associations should not be interpreted as evidence of direct causality. Rather, they suggest that fungi may serve as sensitive indicators of variation in the skin microenvironment (Pianalto et al., 2024).

These findings should be interpreted cautiously given the limited sample size and the exploratory nature of the correlation analysis. Because the microbiome analysis was conducted in a relatively small subset of participants, the present study was not sufficiently powered to draw definitive conclusions regarding microbiome changes after exosome application. In addition, the intervention period may have been too short to induce measurable restructuring of the skin microbiome, as the study consisted of three treatment sessions over six weeks with the final evaluation conducted two weeks after the last treatment. Future studies with larger cohorts, longer follow-up periods, and experimental validation will be necessary to determine whether the observed fungal associations reflect HACS-related barrier improvement or secondary changes in the skin microenvironment.

Despite these limitations, our findings underscore the value of examining both bacterial and fungal communities in skin microbiome studies. Although the overall microbiome response to HACS was modest, the observation that specific fungal taxa, rather than the fungal community as a whole, were significantly associated with multiple skin parameters suggests that fungal signatures may provide complementary information on skin functional status. These results support further investigation of the skin mycobiome in future studies of exosome-based skin interventions.

Taken together, the improvements in hydration, elasticity, barrier function, and wrinkle reduction, along with the modest and largely exploratory changes in the skin microbiome, suggest the potential clinical benefit of HACS as a promising non-invasive intervention for skin rejuvenation. Beyond measurable biophysical and microbial outcomes, consistent physician- and patient-reported improvements further support its translational relevance in real-world dermatological practice. While larger and long-term studies are warranted to confirm these findings, our results provide encouraging evidence that HACS may enhance skin quality and improve patient-reported satisfaction.

## Supplementary Information

Table S1. Facial skin characteristics, including wrinkle depth, pigmentation, sebum level, and brightness, were measured using Mark-Vu facial analysis system. We calculated the mean score for each parameter in both the experimental and control sides, each consisting of 10 subjects. We calculated the change by subtracting the mean score of one side from that of the other side and assessed significant change within each side by evaluating the p-value. We also calculated p-values to determine whether there were any significant differences between the two sides.

Fig. S1. Principal coordinate analysis (PCoA) of bacterial and fungal community composition based on unweighted and weighted UniFrac distances. (A) Bacterial communities were compared across visits within the control and experimental sides. No significant differences were detected by PERMANOVA or dispersion tests. (B) Fungal communities showed the same pattern, with no significant differences across visits in either side.

Fig. S2. Principal coordinate analysis (PCoA) of bacterial community composition based on unweighted and weighted UniFrac distances according to clinical parameters. (A) Bacterial communities stratified by physician-assessed improvement: baseline (pre-treatment), non-improved (PGAIS 1‒2), and improved (PGAIS 3‒5). (B) Bacterial communities stratified by subject-reported satisfaction: baseline, non-satisfied (scores 1‒2), and satisfied (scores 3‒5). No significant differences in community composition were detected by PERMANOVA, and no significant differences in dispersion were detected by dispersion tests.

Fig. S3. Taxonomic composition for each subject, with the control side on the left and the experimental side on the right. (A) Bacteria, (B) Fungi.

Fig. S4. Principal coordinate analysis (PCoA) of fungal community composition based on unweighted and weighted UniFrac distances according to clinical parameters. (A) Fungal communities stratified by physician-assessed improvement: baseline (pre-treatment), non-improved (PGAIS 1–2), and improved (PGAIS 3–5). No significant differences in community composition were detected by PERMANOVA. However, dispersion differed significantly on the control side for unweighted UniFrac distance. (B) Fungal communities stratified by subject-reported satisfaction: baseline, non-satisfied (scores 1–2), and satisfied (scores 3–5). No significant differences in community composition were detected by PERMANOVA. However, dispersion differed significantly on both the control and experimental sides for unweighted UniFrac distance.

Fig. S5. Heatmap of the top 20 bacterial taxa showing nominal correlations with skin biophysical parameters. Colors indicate Spearman’s rho values, with red representing positive correlations and blue representing negative correlations. Black outlines and asterisks indicate associations that were significant at the raw *p*-value level (*p* < 0.05, *p* < 0.01, *p* < 0.001). Only the top 20 taxa with nominally significant correlations are shown. None of these associations remained significant after false discovery rate (FDR) correction (*q* > 0.05 for all comparisons).

## References

[b1-jm-2603020] Abarenkov K, Nilsson RH, Larsson KH, Alexander IJ, Eberhardt U (2010). The UNITE database for molecular identification of fungi-recent updates and future perspectives. New Phytol.

[b2-jm-2603020] Bolyen E, Rideout JR, Dillon MR, Bokulich NA, Abnet CC (2019). Reproducible, interactive, scalable and extensible microbiome data science using QIIME 2. Nat Biotechnol.

[b3-jm-2603020] Boxberger M, Cenizo V, Cassir N, La Scola B (2021). Challenges in exploring and manipulating the human skin microbiome. Microbiome.

[b4-jm-2603020] Callahan BJ, McMurdie PJ, Rosen MJ, Han AW, Johnson AJA (2016). DADA2: High-resolution sample inference from Illumina amplicon data. Nat Methods.

[b5-jm-2603020] Cho BS, Kim JO, Ha DH, Yi YW (2018). Exosomes derived from human adipose tissue-derived mesenchymal stem cells alleviate atopic dermatitis. Stem Cell Res Ther.

[b6-jm-2603020] Domaszewska-Szostek A, Krzyżanowska M, Polak A, Puzianowska-Kuźnicka M (2025). Effectiveness of extracellular vesicle application in skin aging treatment and regeneration: Do we have enough evidence from clinical trials?. Int J Mol Sci.

[b7-jm-2603020] Garlet A, Andre-Frei V, Del Bene N, Cameron HJ, Samuga A (2024). Facial skin microbiome composition and functional shift with aging. Microorganisms.

[b8-jm-2603020] Gentile P, Garcovich S (2021). Adipose-derived mesenchymal stem cells (AD-MSCs) against ultraviolet (UV) radiation effects and the skin photoaging. Biomedicines.

[b9-jm-2603020] Haddad S, Galadari H, Patil A, Goldust M, Al Salam S (2022). Evaluation of the biostimulatory effects and the level of neocollagenesis of dermal fillers: A review. Int J Dermatol.

[b10-jm-2603020] Han JH, Kim HS (2024). Skin deep: The potential of microbiome cosmetics. J Microbiol.

[b11-jm-2603020] Jones KR, Walke JB, Becker MH, Belden LK, Hughey MC (2021). Time in the laboratory, but not exposure to a chytrid fungus, results in rapid change in spring peeper (*Pseudacris crucifer*) skin bacterial communities. Ichthyol Herpetol.

[b12-jm-2603020] Kammeyer A, Luiten R (2015). Oxidation events and skin aging. Ageing Res Rev.

[b13-jm-2603020] Kwon HH, Yang SH, Park BC, Park KY, Jung JY (2020). Combination treatment with human adipose tissue stem cell-derived exosomes and fractional CO_2_ laser for acne scars: A 12-week prospective, double-blind, randomized, split-face study. Acta Derm Venereol.

[b14-jm-2603020] Lee JH, Jeon H, Lötvall J, Cho BS (2024). Therapeutic potential of mesenchymal stem cell-derived extracellular vesicles in SARS-CoV-2 and H1N1 influenza-induced acute lung injury. J Extracell Vesicles.

[b15-jm-2603020] Lee HJ, Kim M (2022). Skin barrier function and the microbiome. Int J Mol Sci.

[b16-jm-2603020] Lee KA, Ul-Haq A, Seo H, Jo S, Kim S (2025). Characteristics of skin microbiome associated with disease severity in systemic sclerosis. J Microbiol.

[b17-jm-2603020] Lee JH, Won YJ, Kim H, Choi M, Lee E (2023). Adipose tissue-derived mesenchymal stem cell-derived exosomes promote wound healing and tissue regeneration. Int J Mol Sci.

[b18-jm-2603020] Lei L, Zhou S, Zeng L, Gu Q, Xue H (2025). Exosome-based therapeutics in dermatology. Biomater Res.

[b19-jm-2603020] Martic I, Jansen-Dürr P, Cavinato M (2022). Effects of air pollution on cellular senescence and skin aging. Cells.

[b20-jm-2603020] Martin M (2011). Cutadapt removes adapter sequences from high-throughput sequencing reads. EMBnet J.

[b21-jm-2603020] Mohiuddin AK (2019). Skin aging & modern age anti-aging strategies. Int J Clin Dermatol Res.

[b22-jm-2603020] Nguyen UT, Kalan LR (2022). Forgotten fungi: The importance of the skin mycobiome. Curr Opin Microbiol.

[b23-jm-2603020] Pageon H, Zucchi H, Rousset F, Monnier VM, Asselineau D (2014). Skin aging by glycation: Lessons from the reconstructed skin model. Clin Chem Lab Med.

[b24-jm-2603020] Park GH, Kwon HH, Seok J, Yang SH, Lee J (2023). Efficacy of combined treatment with human adipose tissue stem cell-derived exosome-containing solution and microneedling for facial skin aging: A 12-week prospective, randomized, split-face study. J Cosmet Dermatol.

[b25-jm-2603020] Pianalto KM, Telzrow CL, Brown Harding H, Brooks JT, Granek JA (2024). *Malassezia* responds to environmental pH signals through the conserved Rim/Pal pathway. mBio.

[b26-jm-2603020] Ratanapokasatit Y, Laisuan W, Rattananukrom T, Petchlorlian A, Thaipisuttikul I (2022). How microbiomes affect skin aging: The updated evidence and current perspectives. Life (Basel).

[b27-jm-2603020] SanMiguel AJ, Meisel JS, Horwinski J, Zheng Q, Bradley CW (2018). Antiseptic agents elicit short-term, personalized, and body site-specific shifts in resident skin bacterial communities. J Invest Dermatol.

[b28-jm-2603020] Saville CR, Metris A, Humphreys GJ, O'Neill C, Barrett P (2022). Transitory shifts in skin microbiota composition and reductions in bacterial load and psoriasin following ethanol perturbation. mSphere.

[b29-jm-2603020] Shibagaki N, Suda W, Clavaud C, Bastien P, Takayasu L (2017). Aging-related changes in the diversity of women's skin microbiomes associated with oral bacteria. Sci Rep.

[b30-jm-2603020] Shin KO, Ha DH, Kim JO, Crumrine DA, Meyer JM (2020). Exosomes from human adipose tissue-derived mesenchymal stem cells promote epidermal barrier repair by inducing de novo synthesis of ceramides in atopic dermatitis. Cells.

[b31-jm-2603020] Shin KO, Lee JH, Chae S, Goto K, An H (2025). Small EVs from adipose-derived MSCs modulate epidermal barrier and inflammation via sphingosine-1-phosphate signaling pathway. J Extracell Vesicles.

[b32-jm-2603020] Szlávicz E, Szabó Á, Kinyő Á, Szeiffert A, Bancsók T (2024). Content validity of the EQ-5D-5L with skin irritation and self-confidence bolt-ons in patients with atopic dermatitis: A qualitative think-aloud study. Qual Life Res.

[b33-jm-2603020] Wang Q, Cole JR (2024). Updated RDP taxonomy and RDP classifier for more accurate taxonomic classification. Microbiol Resour Announc.

[b34-jm-2603020] Wong QYA, Chew FT (2021). Defining skin aging and its risk factors: A systematic review and meta-analysis. Sci Rep.

[b35-jm-2603020] Woo YR, Kim HS (2024). Interaction between the microbiota and the skin barrier in aging skin: A comprehensive review. Front Physiol.

[b36-jm-2603020] Yi KH, Winayanuwattikun W, Kim SY, Wan J, Vachatimanont V (2024). Skin boosters: Definitions and varied classifications. Skin Res Technol.

[b37-jm-2603020] Zhou W, Fleming E, Legendre G, Roux L, Latreille J (2023). Skin microbiome attributes associate with biophysical skin ageing. Exp Dermatol.

