# Supplementary material for: Adipose tissue-derived stem cell exosomes enhance skin barrier function and show exploratory associations with the skin mycobiome in aging skin
Source: J Microbiol. 2026 Jun 30;64(6):e2603020. doi: 10.71150/jm.2603020 (PMC13577115; doi:10.71150/jm.2603020)
Supplement: Supplementary file 1 [file jm-2603020-Supplementary-Fig-S1.pdf]

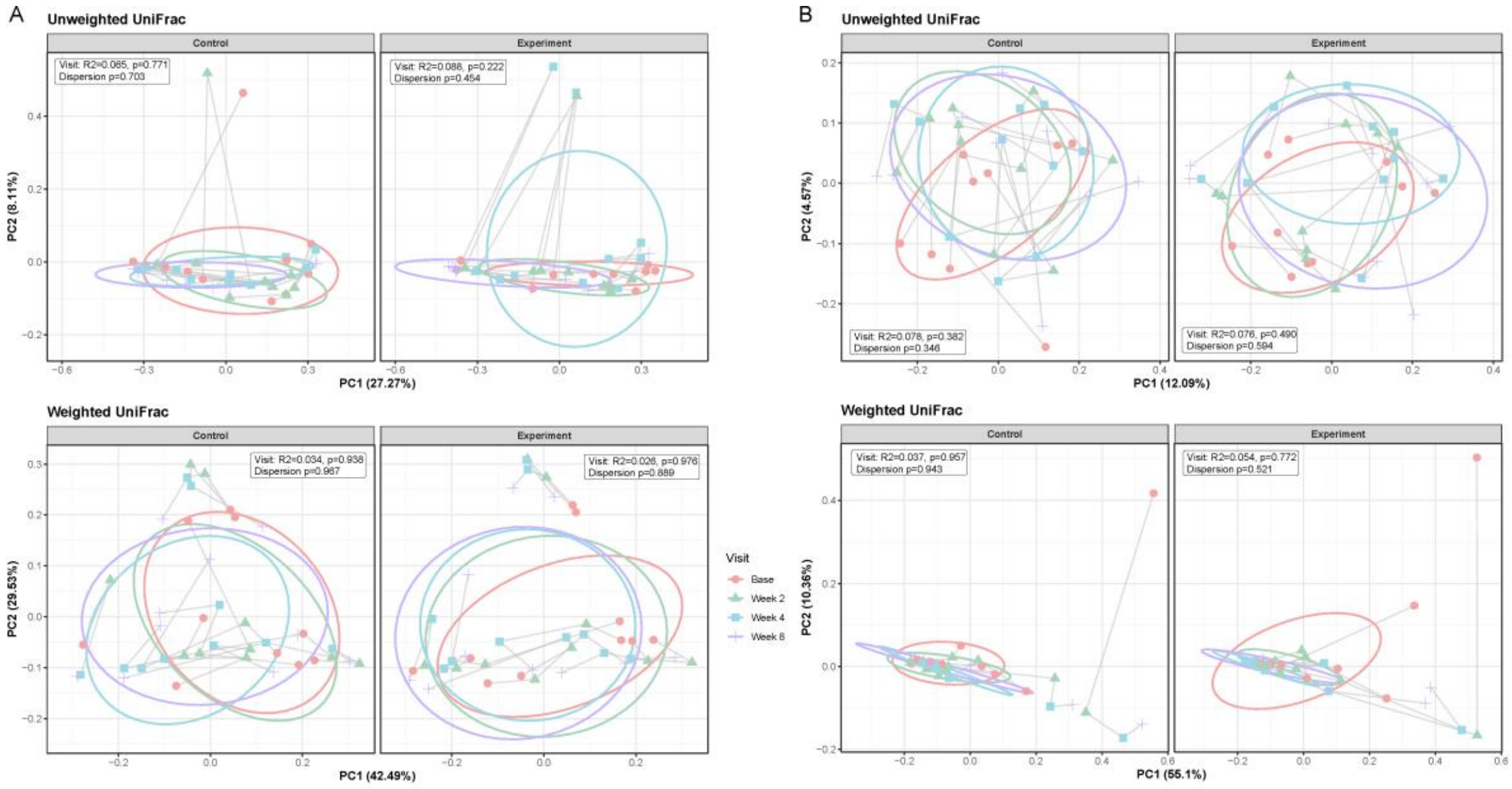

Fig. S1. Principal coordinate analysis (PCoA) of bacterial and fungal community composition based on unweighted and weighted UniFrac distances. (A) Bacterial communities were compared across visits within the control and experimental sides. No significant differences were detected by PERMANOVA or dispersion tests. (B) Fungal communities showed the same pattern, with no significant differences across visits in either side.
