# Supplementary material for: Adipose tissue-derived stem cell exosomes enhance skin barrier function and show exploratory associations with the skin mycobiome in aging skin
Source: J Microbiol. 2026 Jun 30;64(6):e2603020. doi: 10.71150/jm.2603020 (PMC13577115; doi:10.71150/jm.2603020)
Supplement: Supplementary file 2 [file jm-2603020-Supplementary-Fig-S2.pdf]

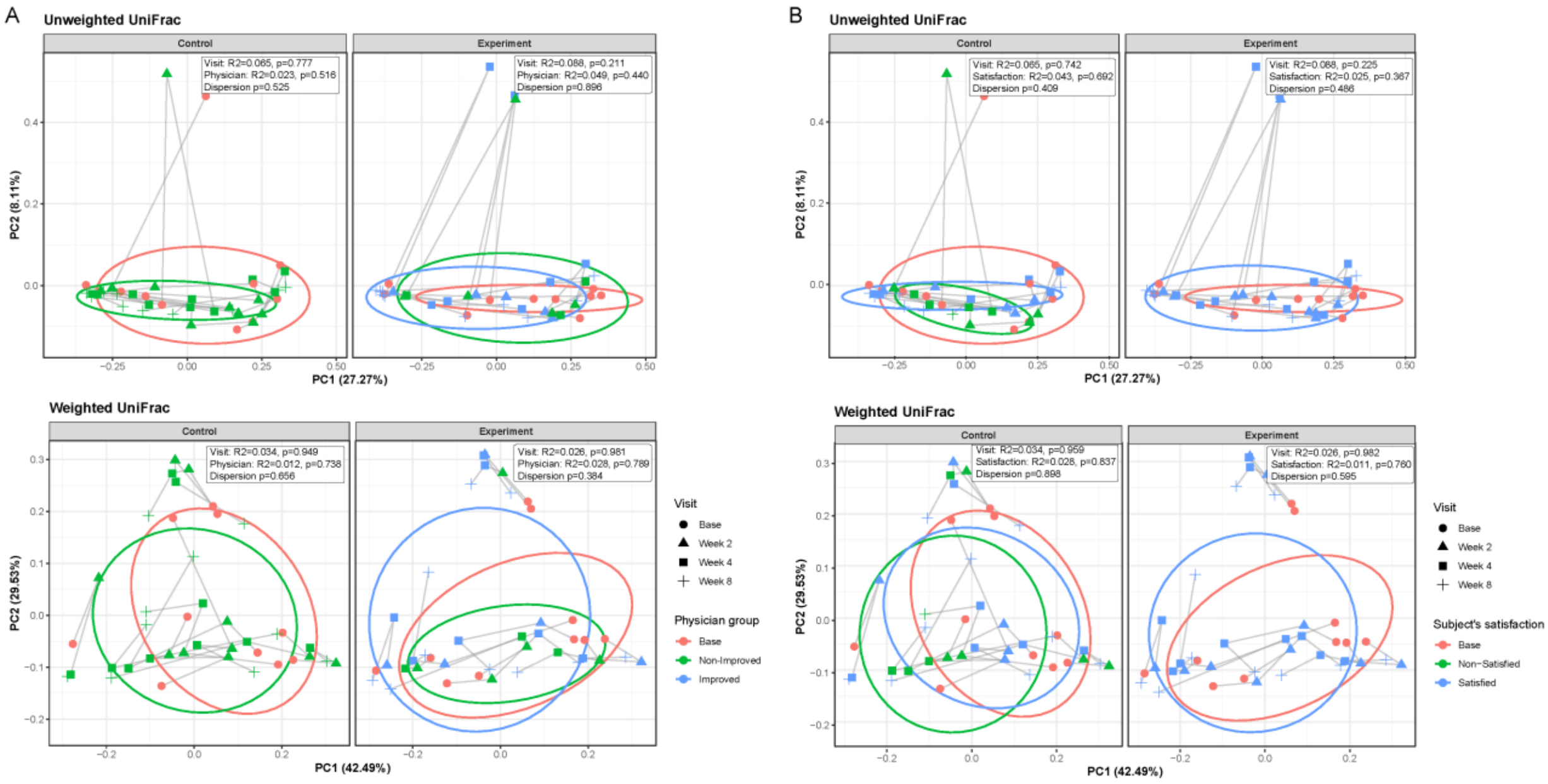

Fig. S2. Principal coordinate analysis (PCoA) of bacterial community composition based on unweighted and weighted UniFrac distances according to clinical parameters. (A) Bacterial communities stratified by physician-assessed improvement: baseline (pre-treatment), non-improved (PGAIS 1–2), and improved (PGAIS 3–5). (B) Bacterial communities stratified by subject-reported satisfaction: baseline, non-satisfied (scores 1–2), and satisfied (scores 3–5). No significant differences in community composition were detected by PERMANOVA, and no significant differences in dispersion were detected by dispersion tests.
