# Supplementary material for: Adipose tissue-derived stem cell exosomes enhance skin barrier function and show exploratory associations with the skin mycobiome in aging skin
Source: J Microbiol. 2026 Jun 30;64(6):e2603020. doi: 10.71150/jm.2603020 (PMC13577115; doi:10.71150/jm.2603020)
Supplement: Supplementary file 3 [file jm-2603020-Supplementary-Fig-S3.pdf]

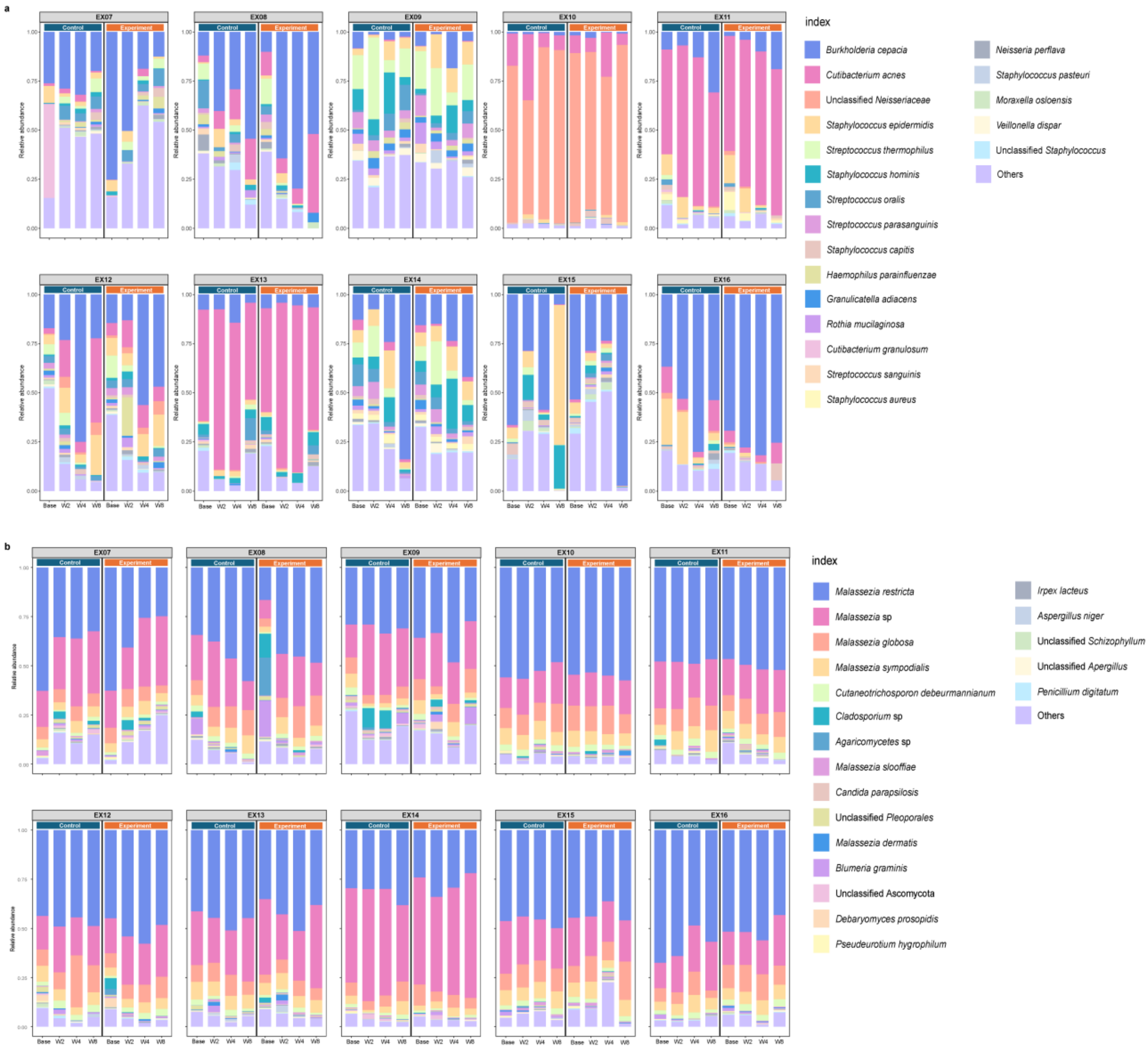

Fig. S3. Taxonomic composition for each subject, with the control side on the left and the experimental side on the right. (A) Bacteria, (B) Fungi.
