# Supplementary material for: Adipose tissue-derived stem cell exosomes enhance skin barrier function and show exploratory associations with the skin mycobiome in aging skin
Source: J Microbiol. 2026 Jun 30;64(6):e2603020. doi: 10.71150/jm.2603020 (PMC13577115; doi:10.71150/jm.2603020)
Supplement: Supplementary file 4 [file jm-2603020-Supplementary-Fig-S4.pdf]

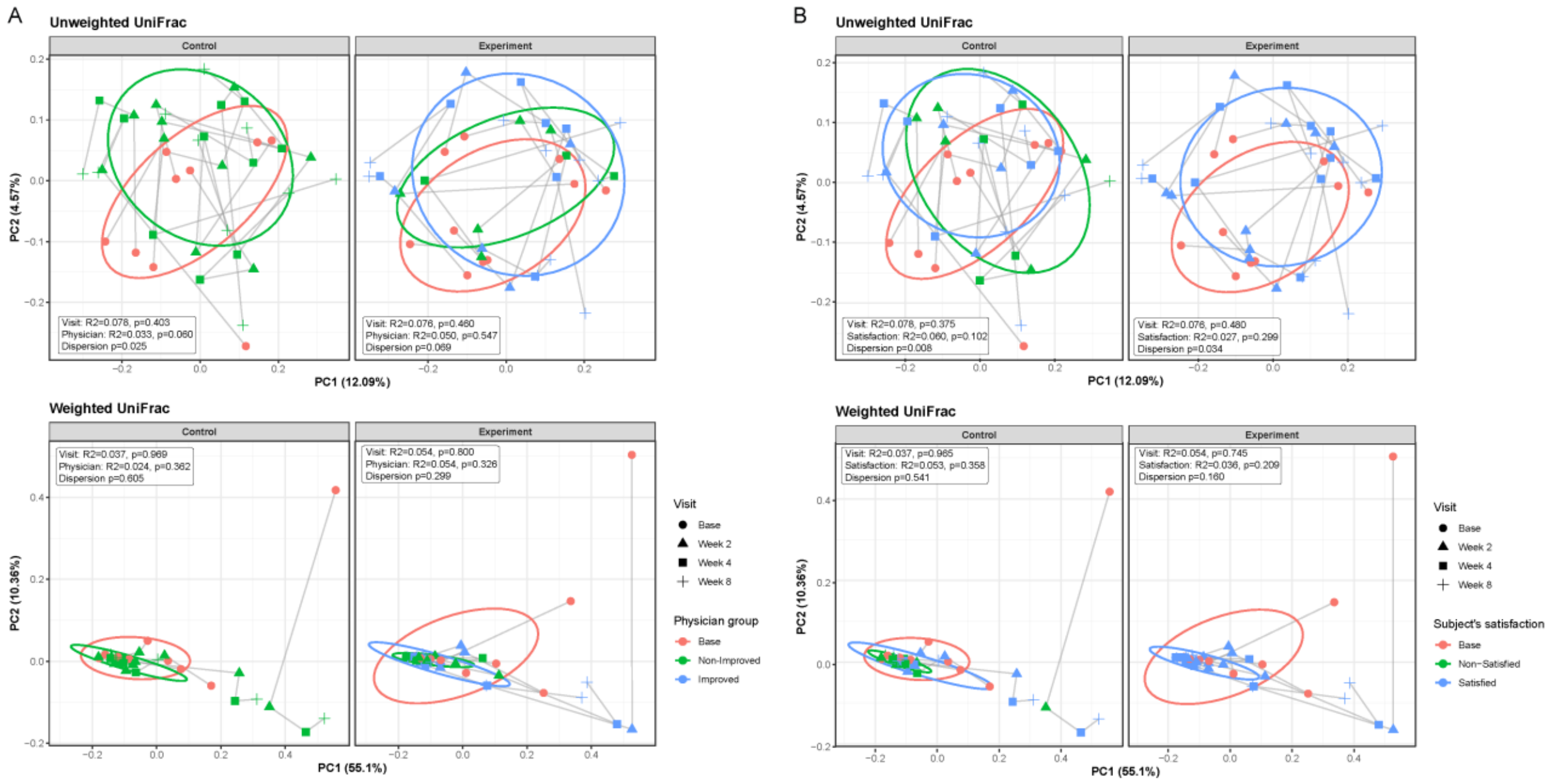

Fig. S4. Principal coordinate analysis (PCoA) of fungal community composition based on unweighted and weighted UniFrac distances according to clinical parameters. (A) Fungal communities stratified by physician-assessed improvement: baseline (pre-treatment), non-improved (PGAIS 1–2), and improved (PGAIS 3–5). No significant differences in community composition were detected by PERMANOVA. However, dispersion differed significantly on the control side for unweighted UniFrac distance. (B) Fungal communities stratified by subject-reported satisfaction: baseline, non-satisfied (scores 1–2), and satisfied (scores 3–5). No significant differences in community composition were detected by PERMANOVA. However, dispersion differed significantly on both the control and experimental sides for unweighted UniFrac distance.
