# Supplementary material for: Adipose tissue-derived stem cell exosomes enhance skin barrier function and show exploratory associations with the skin mycobiome in aging skin
Source: J Microbiol. 2026 Jun 30;64(6):e2603020. doi: 10.71150/jm.2603020 (PMC13577115; doi:10.71150/jm.2603020)
Supplement: Supplementary file 5 [file jm-2603020-Supplementary-Fig-S5.pdf]

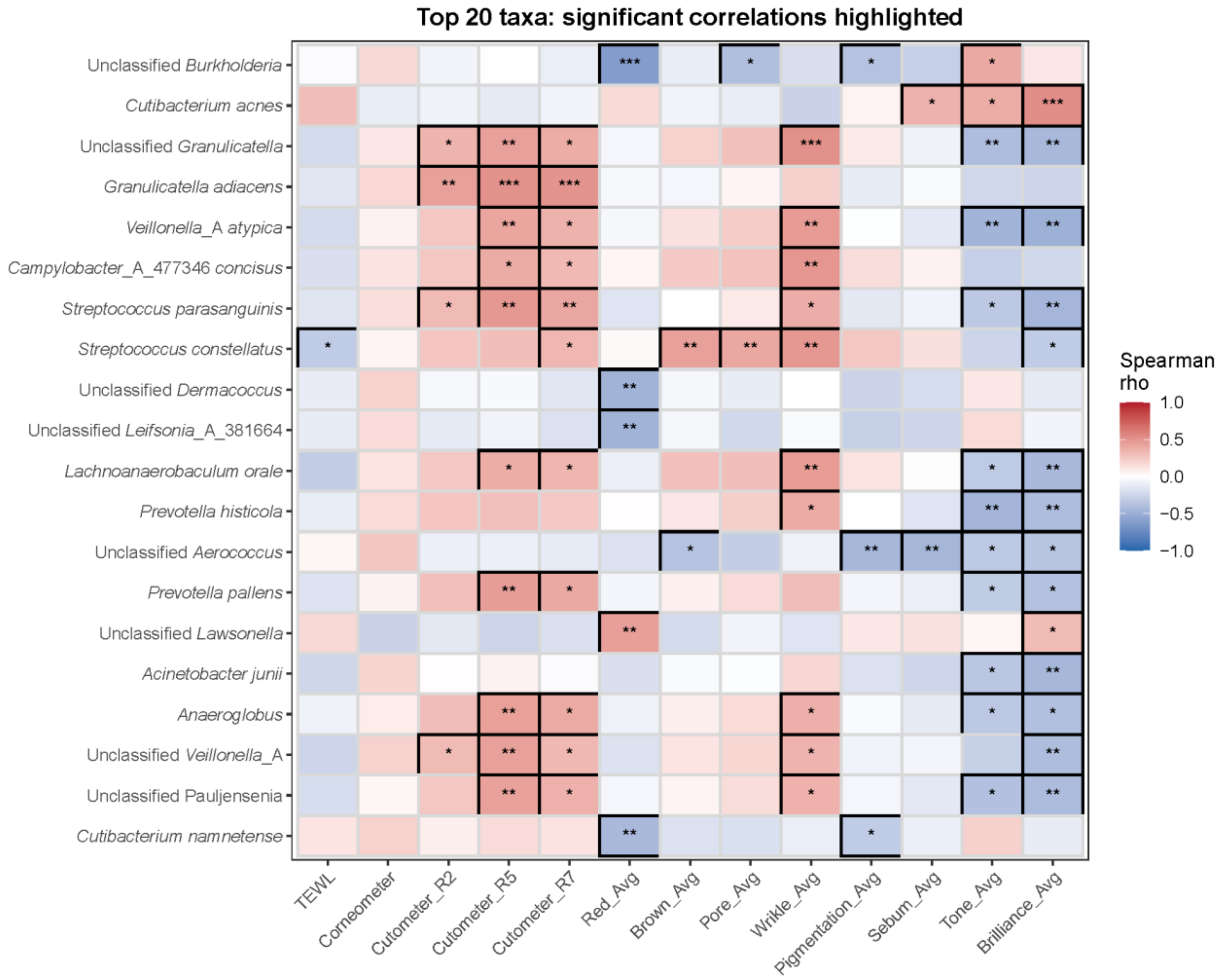

Fig. S5. Heatmap of the top 20 bacterial taxa showing nominal correlations with skin biophysical parameters. Colors indicate Spearman's rho values, with red representing positive correlations and blue representing negative correlations. Black outlines and asterisks indicate associations that were significant at the raw *p*-value level ($p < 0.05$, $p < 0.01$, $p < 0.001$). Only the top 20 taxa with nominally significant correlations are shown. None of these associations remained significant after false discovery rate (FDR) correction ($q > 0.05$ for all comparisons).
