# Supplementary material for: Adipose tissue-derived stem cell exosomes enhance skin barrier function and show exploratory associations with the skin mycobiome in aging skin
Source: J Microbiol. 2026 Jun 30;64(6):e2603020. doi: 10.71150/jm.2603020 (PMC13577115; doi:10.71150/jm.2603020)
Supplement: Supplementary file 6 [file jm-2603020-Supplementary-Table-S1.pdf]

Table S1. Facial skin characteristics, including wrinkle depth, pigmentation, sebum level, and brightness, were measured using Mark-Vu facial analysis system. We calculated the mean score for each parameter in both the experimental and control sides, each consisting of 10 subjects. We calculated the change by subtracting the mean score of one side from that of the other side and assessed significant change within each side by evaluating the p-value. We also calculated p-values to determine whether there were any significant differences between the two sides.

Parameter	Visit	Experiment Mean ± SD	Control Mean ± SD	Exp Δ (Exp - Base)	Ctrl Δ (Ctrl - Base)	p intra (Exp)	p intra (Ctrl)	p inter (Δ)
Red	Base	11.7 ± 4.2	10.4 ± 4.6	-	-	-	-	-
	2weeks	11 ± 3.4	10 ± 3.5	-0.7	-0.4	0.43	0	0.79
	4weeks	10.5 ± 4.1	9.1 ± 3.6	-1.2	-1.3	0.28	0.33	0.76
	8weeks	9.9 ± 3.9	9.7 ± 4	-1.8	-0.7	0.09	0.89	0.34
Brown	Base	22 ± 4.9	23.7 ± 4.7	-	-	-	-	-
	2weeks	22.3 ± 5.7	24.6 ± 5.5	0.3	0.9	0.72	0.23	0.36
	4weeks	22 ± 4.8	24.2 ± 6.1	0	0.5	1	0.62	0.66
	8weeks	21.7 ± 5.4	24.3 ± 5.2	-0.3	0.6	0.73	0.4	0.34
Pore	Base	59.1 ± 3.1	60.7 ± 3	-	-	-	-	-
	2weeks	59.1 ± 4.3	60 ± 4	0	-0.7	1	0.93	0.55
	4weeks	58.4 ± 3.4	60.6 ± 4.2	-0.7	-0.1	0.35	1	0.54
	8weeks	57.7 ± 4.7	60.4 ± 3.4	-1.4	-0.3	0.15	1	0.33
Wrinkle	Base	27.8 ± 1.9	33.5 ± 2.3	-	-	-	-	-
	2weeks	27.6 ± 2	34.5 ± 2.2	-0.2	1	0.83	0.6	0.5
	4weeks	27.7 ± 1.8	34.4 ± 1.8	-0.1	0.9	0.93	0.2	0.19
	8weeks	26.3 ± 2.9	34 ± 1.9	-1.5	0.5	0.04	1	0.09
Pigment	Base	33.2 ± 5.1	33.5 ± 5.9	-	-	-	-	-
	2weeks	33.6 ± 7.1	34.5 ± 6.9	0.4	1	0.55	0.5	0.85
	4weeks	32.5 ± 7.3	34.4 ± 7.8	-0.7	0.9	0.57	0.95	0.4
	8weeks	31.9 ± 6.8	34 ± 6.9	-1.3	0.5	0.28	0.63	0.22
Sebum	Base	11.5 ± 4.5	12.3 ± 5.1	-	-	-	-	-
	2weeks	12.2 ± 5.8	13 ± 6	0.7	0.7	0.48	0.62	1
	4weeks	12.5 ± 3.6	13.4 ± 3.8	1	1.1	0.4	0.28	0.93
	8weeks	11.8 ± 5.9	14 ± 4.8	0.3	1.7	0.89	0.19	0.5
Tone	Base	55.2 ± 2.5	54.7 ± 2.5	-	-	-	-	-
	2weeks	55 ± 2.9	54.9 ± 2.3	-0.2	0.2	0.48	0.57	0.35
	4weeks	55 ± 3.2	54.9 ± 2.8	-0.2	0.2	0.85	0.57	0.65

	8weeks	55 ± 2.4	54.8 ± 2.4	-0.2	0.1	0.59	0.86	0.56
Glow	Base	25.7 ± 8.8	22.1 ± 9.1	-	-	-	-	-
	2weeks	27.3 ± 10.2	23.6 ± 11.3	1.6	1.5	0.36	0.38	0.96
	4weeks	25 ± 9	22.2 ± 8.8	-0.7	0.1	0.81	1	0.71
	8weeks	27 ± 9.5	24.2 ± 8.4	1.3	2.1	0.22	0.23	0.68
